# Exploring perceived effects from loss of PEPFAR support for outreach in Kenya and Uganda

**DOI:** 10.1186/s12992-021-00729-w

**Published:** 2021-07-17

**Authors:** Mary Qiu, Ligia Paina, Daniela C. Rodríguez, Jess A. Wilhelm, Ezinne Eze-Ajoku, Alexandra Searle, Henry Zakumumpa, Freddie Ssengooba, Caroline MacKenzie, Sara Bennett

**Affiliations:** 1 Department of International Health, Johns Hopkins Bloomberg School of Public Health, Baltimore, MD USA; 2 Makerere University School of Public Health, Kampala, Uganda; 3 Ipsos Kenya, Nairobi, Kenya

**Keywords:** HIV/AIDS, Community outreach, Health systems, PEPFAR, Donor transition, Uganda, Kenya

## Abstract

**Introduction:**

In 2015, the President’s Emergency Plan for AIDS Relief undertook policy shifts to increase efficiencies in its programming, including transitioning HIV/AIDS funding away from low burden areas. We examine the impact of these changes on HIV outreach in Kenya and Uganda.

**Methods:**

Qualitative data collection was conducted as a part of a broader mixed-methods evaluation. Two rounds of facility-level case studies and national-level interviews were conducted in Kenya and Uganda, with health facility, sub-national and central Ministry of Health staff, HIV clients, and implementing partners.

**Results:**

In both countries, the loss of outreach support affected community-based HIV/AIDS education, testing, peer support, and defaulter tracing.

**Discussion:**

Loss of external support for outreach raises concerns for countries’ ability to reach the 90–90-90 UNAIDS target, as key linkages between vulnerable communities and health systems can be adversely affected.

**Conclusion:**

Development partners should consider how to mitigate potential consequences of transition policies to prevent negative effects at the community level.

## Introduction

In 2013, the Joint United Nations Programme on HIV/AIDS (UNAIDS) established the 90–90-90 goal where 90% of all people living with HIV (PLHIV) will know their HIV status, 90% of people diagnosed with HIV receive sustained antiretroviral therapy (ART), and 90% of people receiving ART will achieve viral suppression by 2020 [1]. Country governments and the international community mobilized to reach these targets, and in 2019, it was estimated that globally, 81% of all PLHIV knew their status, 82% of those were on ART, and 88% of those on treatment had undetectable levels of the virus [2]. Despite these achievements however, UNAIDS acknowledged at the end of 2020 that the 90–90-90 targets had not yet been met, and that there is considerable work to be done [3].

Across the treatment cascade, numerous barriers remain at each step to accessing testing and treatment, including cost, health system capacity, transport, stigma, lack of knowledge, and fear [4, 5]. Existing evidence demonstrates that community-based services can successfully support clients in bridging these barriers and entering the formal health system, improving retention along the HIV care continuum [6–8]. Retention in care and sustained treatment is in turn a necessary pre-condition for viral suppression, which leads to population-level benefits, such as reduced transmission [9]. UNAIDS recognizes that community-based services are critical to successful HIV programming, and that such strategies need to be at the forefront of national and global HIV responses in order to reach each of the 90–90-90 goals [10]. Furthermore, research suggests that bringing services into the community is critical to reaching marginalized populations and groups that have fewer interactions with the health system [11, 12].

Community-based services related to HIV/AIDS programming include a wide range of activities, with the broad objective of providing services and support to those who cannot access the formal health system. For example, Rhodes refers to HIV outreach as community-based activities seeking to reduce transmission risk among those who are not reached by existing mechanisms [13]. Boeke et al. define community-based services to include defaulter tracing, adherence counseling, and sensitization programs to reduce stigma [8]. For the purposes of this article, we have defined HIV community-based services (herein referred to as “outreach”) broadly as: activities undertaken by health workers, community health workers (CHWs), or volunteers outside of routine medical care to identify, treat, or manage the medical and/or psychosocial needs of HIV-infected individuals and of any community members at-risk for HIV infection.

### Outreach Funding & Global Donor Trends

Historically, across sub-Saharan Africa (SSA), outreach has largely been funded by donors through vertical programs, with limited integration into the local health system and community structures, and with wide heterogeneity across programs within each country [14]. The costs of these programs have rarely been incorporated into domestic budgets, instead operating externally or in parallel with local systems. This creation of parallel structures has been noted to be problematic, with donor funded outreach taking away health care workers (HCWs) from their regular duties or requiring the use of informal cadres from outside the health system. Such activities also need additional resources for training, transport, allowances and per diem, salaries, and logistics management, which are commonly not available through local health budgets. Where donor funding has decreased, the parallel nature of typical outreach services raises concerns for the ability of domestic health budgets to absorb such costs and retain outreach services [14].

Broadly, global trends in development assistance for health (DAH) have stagnated since 2010, from an annualized growth rate of 11.3% between 2000 and 2010 to 1.8% from 2010 to 2016 [15]. Two main global factors are driving this; first, economic and political factors within traditional donor countries have shifted, leading to lower allocations to development assistance in general; second, economic growth in low and lower- middle income status, means that some are losing eligibility under global funding mechanisms, with the expectation for increased domestic resource mobilization. There is concern, however, that many middle-income countries are still unable to fully finance their health systems using domestic resources, resulting in the concept of the “missing middle” [15, 16]. Evidence suggests that these countries have greater levels of out-of-pocket (OOP) spending, reducing access to services and losing the gains that have been made across numerous health focus areas [15].

Specific to HIV/AIDS funding, the President’s Emergency Plan for AIDS Relief (PEPFAR) has been one of the largest funders for HIV/AIDS programming globally since its launch in 2003. In line with global trends in donor financing, starting in 2015, PEPFAR underwent two significant policy shifts with a focus on increasing efficiency of investments [17]. Firstly, while historical PEPFAR programming had evolved to have an emphasis on community-level services, under the new PEPFAR strategy, there was a re-classification of activities to designate “core” vs “non-core” activities. “Core” activities were considered to be those that directly contribute to HIV control (such as combination prevention), and many community-level activities were designated as “non-core” and de-emphasized for funding [18]. Secondly, PEPAR underwent a “Geographic Prioritization” (GP), placing strategic emphasis and resources on sub-national units (SNUs) with the highest burden of disease, targeting “core” activities to these regions [17]. As a part of the GP process, PEPFAR designated SNUs into three key categories to guide priority investments: (1) Scale-Up SNUs receiving increased investment for HIV/AIDS programming, (2) Maintenance SNUs receiving the same level of PEPFAR support, and (3) Central-Support (CS) SNUs transitioning from PEPFAR support to the country government/other donor support. “Low-volume” sites in Scale-up and Maintenance regions that failed to meet a certain threshold of client volume could also be transitioned to government/other donor support [19]. All facilities in CS SNUs or classified as CS were to lose site-level support from PEPFAR, with the expectation that the host government would take over funding services in these locations, including for outreach.

### GP in Kenya and Uganda

A mixed-methods evaluation was conducted in Kenya and Uganda to understand the effects of the PEPFAR GP policy on HIV services and health systems, including exploring effects in CS and Maintenance SNUs. Kenya and Uganda were selected for the evaluation based on how the timing of the GP processes aligned with the study period, and interest from the respective United States Agency for International Development (USAID) Missions to support the study. Brief descriptions of each country’s GP and HIV context are presented in Table 1 and 2. A summary timeline of key events for GP in both countries is presented in Table 3, with both countries beginning GP in 2015.

**Table 1:** Overview of Kenya CS Counties

*Kenya Overview* **90–90-90 Estimates:** 89–77% - no estimate (2015–18) [20] **CS counties (out of 47 total counties)**: Garissa, Isiolo, Lamu, Mandera, Marsabit, Tana River, and Wajir **Geographic Region**: Northeast **Total number of facilities transitioned to CS:** 413 **CS county HIV prevalence estimates (2013)**: < 1–4.9% [21] **Proportion of overall HIV budget funded by PEPFAR (2014)**: 64% [22] **Other context**: Most CS counties were simultaneously deprioritized for HIV programming by the Kenyan Government’s AIDS Strategic Framework 2014/15–2018/19, being classified as “low incidence” (contributing to ≤1% of new HIV infections) [23]. The region is remote, with low population density, and has faced challenges with instability and terrorism [24]. Religious differences with the rest of the country may also influence attitudes and stigma towards HIV/AIDS [25]. The region has also experienced increasing infrastructure development over the past decade, including road construction, a new airport, and a new port, raising potential for evolving patterns of disease transmission as migration patterns change [26–28]. Lastly, devolution also took place 2 years prior to GP (2013), divesting accountability for health programs down to newly formed county governments [29].

**Table 2:** Overview of Uganda CS districts

*Uganda Overview* **90–90-90 Estimates:** 84, 87, 88% (2015–18) [20] **CS Districts (out of 112+ total districts)**: Abim, Amudat, Bulambuli, Kaabong, Kapchorwa, Kween, Luuka, Nakapiripirit, Napak, and Pader **Geographic Region**: North east/east **Total number of facilities transitioned to CS:** 734 [30] **Other sites**: 566 “low-volume” facilities across the country [31] **CS District-Level HIV Prevalence Estimates (2014):** 2.6–10.7% [32] **Proportion of overall HIV budget funded by PEPFAR (2014)**: 55% [33] **Other context**: Historically, funding from PEPFAR in this region has been low, and a number of these districts had been recently established at the time of GP, resulting in very young governments and health systems. (e.g. Luuka and Bulambuli)

**Table 3:** Timeline of key GP events in Kenya and Uganda

Year	Kenya [33]	Uganda [34]
2015	• Introduction of GP to national stakeholders/government agencies in Kenya in October • Initiation of PEPAR GP planning process between October – December • USAID informs IPs, who in turn inform counties	• Introduction of GP to national stakeholders/government agencies in Uganda • Initiation of support withdrawal and simultaneous initiation of rationalization process • Significant numbers of private providers (and some public) have support withdrawn
2016	• Cessation of support begins in January for all CS counties • Completion of GP process by August/September	• Relatively few facilities experiencing withdrawal of support
2017	• Limited support reinitiated to six of seven counties with focus on high volume facilities for six months starting in May	• Significant numbers of facilities in CS districts have support withdrawn in-line with IP contracts ending
	• *Round 1 of study data collection (May–June)* • *Round 2 of study data collection (November)*	
2018	• Withdrawal of short-term support, completing GP process	• Completion of GP process in September

Findings from the main evaluation, presented by Wilhelm et al. [30] and Rodríguez et al. [34] indicated that facilities in CS regions of both countries self-reported cessation of outreach after GP took place. In Uganda, amongst sites that had provided outreach prior to GP, CS facilities were significantly more likely to report discontinuing outreach relative to Maintenance facilities (51.6% vs. 4.1%, *p* < 0.001). In Kenya, both Maintenance facilities and CS facilities reported loss of support for outreach: 39% of CS facilities stopped providing outreach vs. 36% of Maintenance (not statistically significant) [34].

In light of overall reductions in donor funding, whether and how resource-constrained governments could absorb the human or management costs associated with outreach after donor transition remains uncertain. To-date, there has been relatively limited assessment of how countries and health systems cope in the aftermath of donor financing reductions or cessation, in particular for activities that have historically been primarily funded by external sources. Specific to the loss of outreach, a study conducted by Bennett et al. found that in India, the transition of the Avahan program from the Bill and Melinda Gates Foundation (BMGF) to government resulted in a reduction in funding for outreach staff, incentives, and changes to availability of services, in turn reducing engagement with the health system and access to care [35]. Similarly, in a recent study published on the effect of PEPFAR GP in Nigeria, a number of outreach services were reported as being lost after the GP process took place, including defaulter tracking, community-based testing and counselling, and service generation activities [36].

In this paper, we explore how the GP process in Kenya and Uganda affected HIV/AIDS outreach and subsequent access to treatment and care, as experienced by clients and providers in CS and Maintenance SNUs.

## Methods

We chose to use country-level case studies for the purpose of descriptive inference on the effect of donor reprioritization of HIV/AIDS funding, with transferability of findings limited to other countries undergoing similar policy changes [37]. The evaluation focused on USAID-supported facilities at the request of the funder (USAID); however, USAID-supported facilities also represented the majority of transitioning facilities at the time (98% in Kenya, 64% in Uganda). Both countries also presented with unique contextual challenges (devolution in Kenya, and decentralization in Uganda) that provided opportunity for additional learnings related to success of GP. The study employed a mixed-methods research design consisting of five distinct, interrelated components, as presented in Table 4. The importance of exploring how CS facilities and SNUs coped after losing PEPFAR support for outreach emerged as a finding from the first round of qualitative data collection and the facility survey in both countries. We therefore used the second round of key informant interviews (KIIs) and longitudinal facility-level case studies to follow-up more closely on the issue of outreach.

**Table 4:** Study methodology and link to assessing outreach services

Methodology	Sub-component relevant to outreach
Documentation of implementation 1. Document Review 2. KIIs with USG, Ministry of Health (MOH), civil society, and implementing partners (IPs) (2 rounds)	Identification of the specifics and consequences of loss of outreach. KIIs used to triangulate findings from case studies.
Facility Survey^b^ 3. Facility surveys 4. Collection of routine health information system data	Data from the facility survey identified that outreach related services had been affected during the time period of interest^a^
Longitudinal facility case studies 5. In-depth interviews with facility in-charges, district/county-level officials, IP program officers, and focus group discussions with clients (2 rounds)	Facility-level case studies providing local perspectives from the facility and SNU on changes in outreach

^a^Due to the cross-sectional nature of the facility survey, those data are not explored in detail here.

^b^For methods related to the facility survey, please refer to [Author Name Blinded] et al. [35] and [Author Name Blinded] et al. [33]

Although the initial study design and tools were not focused on the effects of outreach specifically, the tools were modified to explore issues around outreach more intentionally for the second round of data collection. In this paper, we draw primarily from the qualitative data collected from the longitudinal facility-level case studies, and focus only on the data relevant to outreach; the main results of the evaluation are presented elsewhere [30, 34, 38, 39].

### Data collection

For KIIs and longitudinal facility case studies we conducted two rounds of data collection in 2017: in May, at 6 months post-transition, and in November at 12 months post-transition. All interviews for both longitudinal case studies and KIIs were conducted in English, while focus group discussions (FGDs) took place in English or the local language of the respective county/district based on respondent preference. For KIIs, we aimed to interview the same respondents in both rounds; however, some respondents interviewed in Round 1 were no longer available in Round 2. New respondents who were identified at the time of data collection for Round 2 were subsequently added. For the longitudinal facility case studies, the same cases facilities visited in both rounds, with the addition of one facility in Uganda (see Table 4).

KIIs at the national level were conducted by SB, DR, LP, and MQ. Qualitative data collection for the longitudinal facility case studies was conducted by local research partners: Ipsos Kenya and Makerere University School of Public Health in Uganda. In both countries, experienced interviewers who spoke local languages and had familiarity with the context of the study SNUs were selected where possible and trained on qualitative data collection. Semi-structured guides were used to allow for exploration of topics that emerged that were not captured in the written questions and probes, and FGDs were conducted with one facilitator and one note-taker. All interviews and FGDs were recorded.

For KIIs, interview guides focused on the process of rolling out GP, timeline, policy development, classification of SNU categories, challenges of implementing GP, perceived effects of GP to date on health services and the health system (including outreach), and future plans linked to GP. Facility case study interview guides focused on respondent experiences with how GP took place, as well as their perceptions on how it had affected HIV and non-HIV service delivery. The second round of case studies was conducted primarily to understand whether facilities had adapted to the GP changes over time. Outreach-related probes were added to the interview guides in the second round of case studies after initial reviews of data, and based on discussions with respective research partners in each country.

### Sampling

KIIs were conducted with a purposively selected sample of national-level Ministry of Health (MoH) officials, United States Government (USG) officials, civil society members, and implementing partners (IPs) in both countries. Participants were identified through a combination of mapping of relevant agencies in each country, and subsequent snowballing during the first round of interviews.

For the longitudinal facility case studies, we purposively selected six case facilities from the overall sample of facilities for the quantitative survey in each country, using facility level (corresponding to facility size and hierarchy of health service delivery in each respective country), transition status (CS or Maintenance), and ownership (public or private) as criteria (Table 5). Facility cases were selected to ensure coverage across geographic regions as it was expected that different SNUs may experience GP differently due to local context and because both countries have decentralized health systems. Stratification across levels ensured representation by size of facility (i.e. tertiary level hospital versus primary care clinic), as it was anticipated that larger facilities may experience GP differently than smaller facilities due to scope of outreach services provided, resource availability, and capacity to adapt. We included at least one Maintenance facility in each country to explore whether outreach had been affected at these facilities in a similar way.

**Table 5:** Selected descriptive characteristics of case facilities

Facility #	County/District	Facility Type*	Ownership	Facility Investment Category
*Kenya*				
1	Garissa	Provincial Hospital	Government	CS
2	Isiolo	District Hospital	Government	CS
3	Mandera	District Hospital	Government	CS
4	Marsabit	Dispensary	Private-not-for-profit	CS
5	Tana River	Health Center	Government	CS
6	Embu	Health Center	Government	Maintenance
*Uganda*				
7	Bulambuli	Health Center	Government	CS
8	Pader	Health Center	Government	CS
9	Luuka	Health Center	Government	CS
10	Kampala	Clinic	Private for profit	CS
11	Amuru	Health Center	Private-not-for-profit	Maintenance
12	Kampala	Hospital	Government	CS
13	Budadiri^a^	Health Center	Government	Inconsistent (CS/Maintenance)

^a^In Uganda, a 7th facility was added after it was found that Budadiri Health Center did not undergo GP, but instead experienced similar effects to transition due to gaps between IP support

For each case facility, we conducted in-depth interviews (IDIs) with the facility in-charge, facility staff, local county/district health officials, local IP program officer, and other relevant individuals, as well as gender-segregated focus group discussions (FGDs) with clients on ART. FGDs were segregated due to the sensitivity of the topic and local gender norms, with each FGD having between 5 and 8 respondents on average. Respondents were identified by convenience sampling those who were attending clinic on the day that the study team visited. All respondents were compensated for their time.

### Data analysis

All interviews and FGDs were transcribed and translated into English, where necessary. Transcripts were uploaded into Atlas.ti 8 for coding. Codebooks were developed a priori based on existing literature, study team experience, and the evaluation’s research questions. The study team double coded initial transcripts and refined the codebook accordingly. Emergent themes were added to the codebook, as needed. While loss of outreach was anticipated, the scope and frequency that this theme appeared was surprising, and codes related to outreach were subsequently expanded and analyzed separately. For KIIs, national-level respondents spoke to outreach at a much broader level, and data was analyzed with the main objective to triangulate what was emerging from the longitudinal facility case study.

For data from the longitudinal case study, outreach coded data were analyzed according to broad categories of community-based services related to HIV treatment and prevention, as defined by the World Health Organization (WHO) [40]. We chose to organize by this framework as the loss of specific types of outreach services was directly linked by respondents to their specific experiences in accessing or providing care, with broader implications to achieving each of the 90–90-90 goals. We modified the existing framework to consolidate categories of services that we felt were similar, resulting in 5 distinct categories: (1) Community-based education, (2) Community-based testing (including case-finding and HIV testing and counselling (HTC)), (3) Peer support networks, (4) Follow-up and defaulter tracing, and (5) Community-based service delivery.

## Results

Across both rounds of data collection, we conducted 62 national-level KIIs with 53 unique respondents across both countries (Table 6). The longitudinal case study included 13 case facilities, with six in Kenya and seven in Uganda. In total, 91 IDIs and 37 FGDs were conducted across all 13 case facilities. We present findings on the experience of outreach and perceived impacts from loss of outreach by the broad categories of community-based services as defined above, followed by data from national-level KIIs that further corroborate the experiences described by respondents at the facility level.

**Table 6:** Characteristics of national-level respondents

	Kenya (***n*** = 19)	Uganda (***n*** = 34)
***Gender***		
M	13 (57%)	23 (59%)
F	6 (26%)	11 (28%)
***Organizational Affiliation***		
IP	6 (26%)	17 (44%)
USG	6 (26%)	6 (15%)
MoH	2 (9%)	3 (8%)
Faith Based Organization (FBO)	3 (13%)	0 (0%)
Civil Society Organization (CSO)	2 (9%)	3 (8%)
Other Donor	0 (0%)	4 (10%)
Other	0 (0%)	1 (3%)

### Community-based education

Loss of support for community-level education on treatment as well as promotion of existing health center services was described as a challenge by several respondents across both Kenya and Uganda. Education programs previously supported under PEPFAR-funded mechanisms were described by providers and SNU staff as focusing on raising overall disease awareness, stigma reduction, encouragement of community members to seek testing and treatment, and to maintain treatment. Clients in FGDs across both countries discussed the types of education sessions they used to receive, reflecting on a range of benefits that were gained from this kind of programming.

*“[IP] used to call us in villages to participate in drama and educate others which also no longer exist. We had bicycles and sometimes transport refund …*” **– Uganda, Client, CS District (Round 1).**

*“We were being taught about the issue of adherence, we learned the importance of doing disclosure with family members...If you have small children and husbands, they were telling us to bring them to be tested.”* **– Kenya, Client, CS County (Round 1).**

In Kenya, respondents from IPs spoke specifically on how they were no longer able to provide educational sessions in the community, and how this affected their ability to generate community-level awareness. One county official described how this was problematic due to stigma in the community against HIV/AIDS, and the ongoing need to address this.

*“HIV is not like any other disease. It is associated with stigma and it is affecting the youth...it is a disease which requires engagement of all stakeholders. Without a partner, running HIV program as a County will be a challenge … especially in terms of community engagement …” –* **Kenya, County Health Officer, CS County (Round 1).**

In Uganda, a small number of facility providers focused specifically on linking lower service volumes and poor treatment continuity with the disruption to education at the community level for both HIV and non-HIV care. One facility provider expressed concern that a lack of community-level education could lead to increases in treatment resistance in the future.

*“We are seeing that there will be resistance, HIV increased treatment failure among clients, we are anticipating many clients getting lost to follow-up. We are anticipating an increased HIV positivity rates because there are no community interventions to reach them and give them the information and the preventive methods.”—* **Uganda, Facility In-Charge, CS District (Round 1).**

Overall, community education for HIV/AIDS appeared to be highly valued by respondents, in particular clients, at all levels for its importance in raising awareness, encouraging community members to be tested, and getting HIV-positive individuals connected with the health system.

### Community-based testing

In both countries, respondents spoke at length during both rounds of case studies as to how their ability to find cases in the community has been reduced due to the end of support for outreach. Previously, outreach to the community allowed them to identify new cases of both HIV and tuberculosis (TB), but health facility staff reported seeing fewer new cases than before as they now relied on the client to come to the clinic for testing. In Kenya, this was reported in CS and Maintenance counties. Kenyan respondents also described how community-based testing had previously helped to overcome barriers to accessing tested.

*“This is a community where stigma is very high. So, convincing somebody to undergo HIV test [ing] here, it is not an easy thing. So, if you do not conduct outreach, the number of clients who are going to come to VCT [voluntary counseling and testing] is very low. So, coverage has gone down.”* – **Kenya, County Health Officer, CS County (Round 1).**

*“We used to go for outreaches during time [convenient to the clients] but now when they [IP] withdrew we don’t do mobile outreach for the HIV testing. We used to do moonlight HIV testing at night, but nowadays it is not conducted.”* **–Kenya, Facility In-Charge, CS County (Round 2).**

In Uganda, respondents described specific barriers that prevented individuals from going to the clinic for testing, including transport challenges, and how clients may be more likely to undergo testing when they are in their own community. One respondent in Uganda also described how outreach allowed them to target key populations (KP) for testing, such as sex workers and fisherfolk, and that they were now unable to do so due to lack of financial support.

“*… now we were not reaching [undiagnosed clients] as we used to because we would go for an outreach and you get like three to five. But now we just wait for those ones who come here to the facility.”* – **Uganda, Facility Staff, CS District (Round 1).**

*“We used even to go in bars at night looking for these sex workers … we used to bring them to us, or we organize seminars we go and address them at night, but now all that stopped … there is nothing being done compared to when [IP] was here … we also used to go to particularly boda-boda people [and] fishermen people, but now you can’t go there, who would pay for transport …”* —**Uganda, Expert Client, CS Facility, Round 1.**

Across both countries, facility respondents consistently described concerns of reductions in coverage of the surrounding community as a result of losing the ability to seek out new clients in the community. One local health official in Uganda also expressed concern on their ability to maintain accurate information on prevalence of the surrounding catchment population without support from ongoing monitoring.

### Follow-up and defaulter tracing

Defaulting from treatment and loss-to-follow-up (LTFU) was one of the most cited concerns in both Kenya and Uganda, and appeared to have been experienced in very similar ways. Respondents extensively described the extent of support that was previously provided by IPs for defaulter tracing, and how loss of this affected the ability of providers to follow-up with clients in the community.

*“At that time, you know we were dealing with HIV patients and there were a lot of defaulters from treatment … there were funds [ …*] *we could go for the clients and find them and bring them back to the facility for treatment”* – **Kenya, Facility In-Charge, CS County (Round 1).**

*“USAID support came up to 2015 and we were particularly supporting HIV activities, ART where they would support follow-up of clients … and follow-up of lost children” –***Uganda, Facility Staff, CS District (Round 1).**

Respondents described both the loss of financial support for allowances, airtime, and fuel for transport, and the loss of informal health workers (such as peer mentors, expert clients, and mentor mothers). Without the support and resources, respondents in both countries described how it was no longer possible to conduct follow-ups, with one facility in-charge in Uganda linking loss of follow-up to client deaths.

*“But now the funds are not there, when a client defaults you cannot reach them. If her phone is not going through you can’t go there because there are no funds.”* – **Kenya, Facility In-Charge, CS County (Round 1).**

*“Right now we have a very big workload because [expert clients] went away … so the workload is now high and that’s why you see tracing for clients is hard, that’s why indirectly there are deaths, some are dying.”* – **Uganda, Facility In-Charge, CS District (Round 2).**

Respondents also highlighted the specific issue of following-up on children who test positive and do not come back to the clinic. Previously, there were mechanisms to follow-up with mothers at home, but this was not maintained after GP.

*“… we had the structure of the mentor mothers who were so vital to the HIV structure that they could support the clients with follow-up and would link them [to services] and [ …] the positivity in children was going low … Even that follow-up of clients was lost.” –* **Uganda, Facility In-Charge, CS District (Round 1).**

*“In case this child turns positive [ …] the [PI] used to follow-up the mothers at home [ …] to bring the child for enrollment. But right now, nobody is there to support these staff to go to look for those mothers when their children turn positive, so they end up missing.” –***Kenya, Health Records Officer, CS County (Round 1).**

In general, support for follow-up and defaulter tracing appeared to be highly valued by both providers and HIV-positive clients in both countries. Inability to continue follow-up with clients was perceived by respondents to have direct effects on health outcomes, with some respondents even attributing client deaths to loss of follow-up.

### Peer support networks

Loss of PEPFAR-funded peer support groups and/or treatment support groups was more frequently described by respondents in Kenya where the main program previously supported “expert clients” in six out of seven CS counties. Respondents discussed how expert clients led psychosocial groups where HIV-positive clients were able to meet monthly to support each other and share challenges.

*“It used to happen in the morning before we were given the drugs. We would meet and do introductions, we would know each other, we would share challenges.” —***Kenya, Client, CS County (Round 2).**

Respondents in Kenya described how new clients were better able to relate to other clients who had experienced the same thing. With the loss of funding to continue this programming, one county health officer connected this to a decrease in uptake of key health services.

*“These patients they understand more when their fellow [patient] is just talking to them. Rather than me standing there and just telling them ABCD … But somebody who has experience of the drugs they can really listen to her. So she was telling them everything, the lady who was here as the expert patient.”* **–Kenya, Facility Staff, CS County, Round 1, Kenya.**

*“There are those PLHIV support groups that were being supported, that support has ended. So it has actually affected the key uptake of key services.”* **–Kenya, County Health Officer, CS County, Round 1, Kenya.**

In Uganda, the issue of peer support was discussed more specifically in the context of mentor mothers during Round 2, who had previously assisted with retaining children in treatment by encouraging mothers to bring their children to the clinic. One district health official described how they previously provided support groups for mothers that helped them keep track of appointments while providing guidance on how to care for their children.

*“… because we are not following these mothers, these mothers are now not in a group where they know what to do and which month they’re supposed to come and what to do with their children.” —***Uganda, District HIV Focal Point, CS District (Round 2).**

Although more predominantly seen from respondents in Kenya, overall, respondents in both countries reiterated similar themes on how previously IP funded peer support groups provided forums for support and knowledge sharing, and that the loss of this has implications for retention, follow-up, and awareness generation.

### Community-based service delivery

Respondents at various levels also brought up the disruptions to general community-based services, and how this has further affected the HIV care continuum. In Uganda, respondents described how facilities previously were able to provide services such as home delivery of medicine through informal health workers. This appears to have ceased when support for outreach was terminated. One respondent in Uganda in Round 1 described how TB treatment and follow-up services had been affected, while another in Round 2 also described how they used to piggyback malaria treatment delivery and family planning services with HIV outreach.

*“… The [TB] treatment success rate has reduced … we are seeing that clients are not getting cured. Although not yet confirmed but it’s a suspected MDR now. It’s also increasing because of [lack of] follow-up. We used to have community treatment supporters who would take the medicines to the communities but right now we wait for clients to come to the facility.” —***Uganda, Facility In-Charge, CS Facility (Round 1).**

In Kenya, respondents highlighted how CHWs previously encouraged expectant mothers to attend facilities for birth while conducting outreach, which allowed them to also initiate prevention of mother-to-child transmission  (PMTCT) services, but without CHWs they were seeing fewer women delivering in facilities.

*“In terms of facility utilization, bringing the mothers from the community to deliver in the facility, that has reduced … we had a pool of community health volunteers and mentor mothers who we used to support. We had to part with them so we did not have the foot soldiers in the community to bring [mothers to the facility].” —***Kenya, IP Staff, CS District (Round 2).**

In general, loss of support for other community-based services were described as being linked to loss of support for outreach, whereby informal health workers were previously able to leverage HIV funded outreach to provide additional information and support for non-HIV related care while working in the community.

### Triangulation with National-Level Respondents

National-level respondents in both Kenya and Uganda spoke similarly to the loss of outreach at a much broader level compared to facility respondents. They corroborated the loss of outreach services that emerged from the facility case studies, although with fewer details as to how this was experienced, highlighting an overall disconnect between those who work at the national policy level versus providers and clients at the facility level. In Kenya, a representative from a Civil Society Organization (CSO) noted that ART coverage had remained, but the supporting services to ensure that clients can access and use ARTs had not.

*“… ARVs are supposed to go with a package of educational services, training and so on and so forth, that is what is not available. So, if you are talking about ARVs then the coverage stays the same, but if you are talking about these other services that go with ARVs then the scope has narrowed” –* **Kenya, CSO (Round 2).**

Of those at the national level who spoke of outreach, there was an acknowledgement that without these services ongoing, there would be an effect on access to care. In Kenya, a national level IP staff member noted that they had already seen cascading effects on the ability of clients to access both HIV and non-HIV services, while in Uganda, there was an acknowledgement that clients were being lost.

“*So the major effect [was] when we had to drop the community health strategy and structures because that had a huge knock-on effect in terms of better access to HIV services and reproductive, maternal, and newborn child services*”. **– Kenya, IP (Round 1).**

*“Those are the big challenges we are facing, we have [clients] lost- to- follow up because those people were being facilitated, now they are not. So, the onus is on the patients themselves but some patients need some pushing and some supervision and all that work cannot be done by health workers” –* **Uganda, AIDS Control Program (Round 1).**

Respondents broadly reflected on the need and importance of outreach services, and the important role that such programs play in the overall strategy to prevent and treat HIV/AIDS. There was acknowledgement that without support from donors, it was unlikely that such programs could continue, and that quality of services would likely decrease.

“*I may know that the ministry is responsible for taking care of the services of some in the community but if the ministry can also only do so much, then there’s very little they can do.” –* **Uganda, National Level IP Staff (Round 1).**

## Discussion

To date, there has been extensive research conducted around the effects of community-based HIV services across the treatment cascade. Such research has found, with some consensus, that community and home-based care may increase rates of testing for HIV, and support sustained uptake of HIV treatment [6, 41–45]. In the first-year evaluation of the CIRKUITS program in Zambia, targeted testing in the community showed success in reaching men, with high positivity yield (18%) and high linkage rates to treatment (97%) [46]. Similarly, outreach to enhance the impact of “test and treat” for HIV were found to achieve high cascade coverage by the second year of follow-up in 16 communities in Kenya and Uganda, including for men and mobile populations [47]. Outreach components in these studies included community-based multi-disease prevention strategies, home-based testing, facilitated linkage to care, and tracking for individuals who were not linked to care [10, 47].

Our findings indicate that recent shifts in PEPFAR funding and strategy for HIV/AIDS programs in Kenya and Uganda, specifically the transitioning of certain facilities and jurisdictions to CS, resulted directly and indirectly in a loss of financial and technical support for outreach. Based on study findings, local health systems were unable to re-hire, replace or absorb informal staff or reinvigorate community-based programs that had been established with the support of PEPFAR, despite official expectations. According to our respondents, the loss of support for outreach had direct and immediate effects on the ability of facilities to provide certain services. Respondents of all types in both countries consistently indicated that CS facilities have experienced challenges in (i) conducting general education and awareness campaigns to their surrounding communities, (ii) providing testing outside of their facilities, including amongst target populations, (iii) tracing clients who default, (iv) providing clients with psychosocial support through expert clients, peer mentors, and mentor mothers, and (v) facilitating non-HIV related services within the community.

Our respondents’ concerns about how the loss of outreach may affect PLHIV and their communities over time links closely to existing data on the value of outreach services across the treatment cascade in support of reaching the 90–90-90 targets. In relation to the **1st 90**, community-based testing, across modalities, has been shown to achieve high coverage and higher testing rates across KPs compared to facility-based testing [42]. Testing in the community has also been found to identify HIV-positive individuals earlier on in disease progression, allowing for earlier linkage to treatment. Although the shift by PEPFAR to classify community-based testing as a non-core activity is intended to better maximize resources by testing targeted populations rather than populations with very low prevalence, this potentially overlooks the benefit that can be achieved from conducting HIV testing in the broader population [42]. In addition, community-based education and awareness programs have been demonstrated to improve overall knowledge about HIV and its transmission, increasing the proportion of people who get tested and seek counselling [48]. The loss of outreach subsequently severs this initial first linkage between the health system and the community.

For the **2nd 90**, loss of support in the form of expert clients, peer mentors, mentor mothers, and PLHIV support groups has implications for the ability of facilities to initiate and maintain clients on treatment. Psychosocial support through support groups and expert clients or peer mentors has been found to be a significant factor in treatment adherence across studies in SSA, including in Kenya, Swaziland, Lesotho, Namibia, Botswana, Uganda, Malawi, and Ethiopia [49–52]. In addition, the ability to physically trace clients in the community who have defaulted has been linked to better adherence outcomes, even amongst mobile and at-risk populations in unstable settings [51, 53]. Our study context in Kenya, where clients reside in remote and unstable regions of the Northeast, may be particularly susceptible to defaulting as a result of environmental factors, like drought and violence, and may be the most in need of additional support such as defaulter tracing.

Finally, for the **3rd 90**, suppressing viral load is contingent on the ability of clients to maintain sustained treatment. In a multi-faceted community-based program implemented in Kenya and Uganda that included universal testing, linkage to care, treatment, and community-based follow-up, viral suppression increased by 35% over the 2-year intervention [47]. Authors attribute high rates of community engagement and retention partly to the delivery of non-HIV related services for diseases such as hypertension and diabetes, which may have increased the attendance of men. In addition, monitoring of viral load suppression is a critical component for managing treatment and treatment failure in clients; suggested strategies for higher risk populations include testing through mobile clinics at the community level [54, 55]. The loss of support for outreach may limit providers’ ability to monitor clients on a routine basis, in turn increasing risk for treatment failure, treatment resistance, transmission, and morbidity.

In losing support for outreach without adequate replacement by local/national governments or other donors, there is concern that a critical linkage into communities will be lost and exacerbated by existing stigma, changing migration patterns and ongoing health system decentralization. In Kenya, new infrastructure development brings concerns for increased migration and evolving patterns of disease transmission, in an ongoing setting of instability due to conflict and violence [26, 27]. In Uganda, some districts categorized as CS were established relatively recently, split from districts where HIV prevalence and burden are high (e.g. Luuka and Bulambuli), and poor governance and weak health systems may continue to pose a challenge. Similarly, in Kenya, devolution to the county system took place in 2013, only 3 years prior to when GP began. In both countries, the loss of support for outreach may be complicated by reduced monitoring in settings where the health system is weak, geographic barriers exist to reaching facilities, and in the case of Kenya, ongoing violence in the transitioned region complicates access. At the time that this study was conducted, neither country had yet to identify a strategy or plan to account for the loss of PEPFAR support to ensure the continuation of outreach, nor does it seem that the contextual issues and emerging trends in the HIV epidemic were considered for the decision to terminate support.

### Lessons learned

There are a number of lessons that can be learned from the experiences documented in Kenya and Uganda for future funding transitions in HIV programming as well as other disease areas. Firstly, improved communication down to the sub-national and facility level may have provided opportunity for existing outreach programs to be better integrated into local budgets, or for alternative donors to be identified. In Kenya, it was found that although GP was well communicated at the national level, there was little to no communication down to the counties, resulting in abrupt cessation of services at the facility level [34]. In Uganda, while the majority of facilities were directly informed by IPs, on average they were provided with 3 months-notice, which was likely insufficient for facilities to identify feasible ways to maintain their outreach programs [56]. Secondly, in both countries, the overall timeline for GP was relatively short. GP planning first began in 2015, and was largely completed by late 2017. Furthermore, GP took place in both countries outside the normal budget cycle, limiting opportunities for restructuring of funding to cover outreach. The rapid pace at which this took place, coupled with limited communication and documentation, likely resulted in a missed opportunity for local actors and government to create a cohesive plan to step-in [56]. Thirdly, ongoing support for monitoring would be valuable for assessing changing needs over time as the local health system attempts to adapt. In Kenya, the IP stepped in for a period of 6 months to reinstitute support after monitoring data indicated that counties were not able to cope [34]. In Uganda, the frequency of respondents describing patients LTFU is concerning, and ongoing monitoring support may be able to detect shifts in HIV service provision. These lessons align with what Vogus and Graff describe as “six key steps for transition,” regarding the need for intensive stakeholder involvement over a prolonged period, and the importance of ongoing support for monitoring and evaluation to ensure minimal disruption of services [57].

More broadly, PEPFAR’s GP process reflects the need for development assistance partners to reflect critically on the outsized role they have played in shaping country responses to the HIV epidemic over the decades and to take this into consideration when planning transition efforts. Foreign aid and development assistance for health has heavily influenced health programming, and some argue that DAH has detracted from country level efforts to invest in building up robust health systems, instead prioritizing spending to align with donor objectives and short-term projects [58]. Donor funding for HIV, in many places, has created parallel health systems while siphoning off critical human resources to externally funded projects [59]. As seen in the findings presented, these gaps are subsequently exposed when long-term funding disappears, leaving fragile and poorly funded health systems to adapt under severe resource constraints. In the global context of decreasing funding, donors should consider that they have had a disproportionate amount of influence in shaping country health systems, and must take responsibility for ensuring that any reductions to funding are done so in a way that does not do further harm. This responsibility is particularly important with regards to avoiding potential harms for affected communities and individual patients who cannot engage in global dialogues on DAH.

There is a need for further research on how losing existing outreach impacts not only immediate health service delivery but also client physical and mental well-being, disease patterns, and epidemiology in the longer term, particularly for KPs and marginalized populations. Further study into how outreach services have or could rebound after transition are also needed to help with successful transition planning. Finally, research and experimentation to understand how integrated outreach efforts may be carried out across services, such as TB, maternal, neonatal, and child health, or other chronic diseases is critical to identifying ways in which such services can be maintained without the same levels of donor support. As global funding levels stagnate, such research is needed to ensure that countries can adequately achieve global targets for ending the HIV epidemic and achieving the UNAIDS 90–90-90 goal.

### Limitations

This study presents a number of limitations. Firstly, outreach was not a key focus of the original evaluation but the loss of support and the attendant effects emerged as an important theme across data sources early on. Secondly, in both countries, other ongoing events at the national level took place during the study period, possibly affecting respondents’ views. In Kenya, two health worker strikes caused notable service disruption across the country during the study period making it difficult, at times, to reach relevant respondents. In Uganda, the process of rationalization whereby districts were transferred between PEPFAR agencies (e.g. USAID, the Centers for Disease Control and Prevention, and the Department of Defense) in an attempt to improve overall efficiency and avoid duplication took place simultaneously with GP. Respondents were often confused by the two processes due to their similarity, potentially affecting their recall. Thirdly, it is possible that respondents may have overstated the effects of losing outreach services in the hope that this would lead to a resumption of support, however interviewers introduced themselves in a manner which did not associate them with potentially influential stakeholders such as USG. Fourthly, while no respondents refused to speak during the facility level case studies, a number of invited FGD respondents did not come to the actual focus group. Key voices and experiences at the client level may therefore have been missed. Finally, this study took place in the period immediately following GP, with relatively short periods of time between rounds. It is possible that the effects of the GP had yet to be fully experienced, and additional follow-up research may elucidate longer term effects.

## Conclusions

This article provides early explorations on how the loss of support for outreach has potential implications across the HIV care continuum. While the findings from this study cannot estimate long-term effects, evidence on the positive effect of outreach services on outcomes linked to the 90–90-90 goals raises concerns for communities that have been transitioned away from PEPFAR support without sufficient replacement of services. Outreach services act as a key linkage between communities and the health system, in particular for the most vulnerable populations and populations that are geographically isolated, such as the sites included in this study. In regions with low prevalence and incidence rates of HIV/AIDS, it is important to retain efforts to prevent changes to the epidemiology of the disease through ongoing education, and maintaining links between the health system and the community. Future donor transitions should ensure engagement with the local health systems, sufficient timelines to prepare and identify alternative funding for outreach, and commit to supporting M&E to capture shifts in service provision and disease patterns. As countries work toward achieving the 90–90-90 goals in the context of declining donor funds and a greater need for efficiency, policy-makers should continue to consider the longer-term benefits of supporting outreach programs.

## Data Availability

The qualitative datasets generated and/or analysed during the current study are not publicly available as this would breach confidentiality.

## Abbreviations

ART: Antiretroviral therapy
BMGF: Bill and Melinda Gates foundation
CHW: Community health worker
CS: Central support
CSO: Civil society organization
DAH: Development assistance for health
FGD: Focus group discussion
GP: Geographic prioritization
HTC: HIV testing and counselling
IDI: In-depth interview
IP: Implementing partner
KII: Key informant interview
KP: Key populations
LTFU: Loss-to-follow-up
MoH: Ministry of health
PEPFAR: President’s emergency plan for AIDS relief
PLHIV: People living with HIV
PMTCT: Prevention of mother-to-child transmission
SSA: Sub-Saharan Africa
SNU: Sub-national unit
TB: Tuberculosis
UNAIDS: Joint United Nations Programme on HIV/AIDS
USAID: United States Agency for International Development
USG: United States government
WHO: World health organization

## References

[1] UNAIDS (2014). 90–90-90: an ambitious treatment target to help end the AIDS epidemic.

[2] UNAIDS. Global HIV & AIDS Statistics- 2020 Fact Sheet 2020.

[3] Levi J, Raymond A, Pozniak A, Vernazza P, Kohler P, Hill A (2016). Can the UNAIDS 90-90-90 target be achieved? A systematic analysis of national HIV treatment cascades. BMJ Glob Heal.

[4] Boender TS, Sigaloff KCE, Kayiwa J, Musiime V, Calis JCJ, Hamers RL, Nakatudde LK, Khauda E, Mukuye A, Ditai J, Geelen SP, Mugyenyi P, Rinke de Wit TF, Kityo C (2012). Barriers to initiation of pediatric HIV treatment in Uganda: a mixed-method study. AIDS Res Treat.

[5] Crooks D, Fox MP, Mazimba A, Seidenberg P, Rosen S, Sikateyo B (2010). Barriers to initiation of antiretroviral treatment in rural and urban areas of Zambia: a cross-sectional study of cost, stigma, and perceptions about ART. J Int AIDS Soc.

[6] Govindasamy D, Meghij J, Negussi EK, Baggaley RC, Ford N (2014). Interventions to improve or facilitate linkage to or retention in pre-ART (HIV) care and initiation of ART in low- and middle-income settings – a systematic review. J Int AIDS Soc..

[7] Bean MC, Scott L, Kilby JM, Richey LE (2017). Use of an outreach coordinator to reengage and retain patients with HIV in care. AIDS Patient Care STDs.

[8] Boeke CE, Nabitaka V, Rowan A, Guerra K, Nawaggi P, Mulema V, Bigira V, Magongo E, Mucheri P, Musoke A, Katureebe C (2018). Results from a proactive follow-up intervention to improve linkage and retention among people living with HIV in Uganda: a pre−/post- study. BMC Health Serv Res.

[9] Yehia BR, Fleishman JA, Metlay JP, Moore RD, Gebo KA (2012). Sustained viral suppression in HIV-infected patients receiving antiretroviral therapy. JAMA..

[10] UNAIDS. Ending AIDS: Progress towards the 90–90-90 targets. Geneva: UNAIDS; 2017.

[11] Staveteig S, Croft TN, Kampa KT, Head SK. Reaching the ‘first 90’: Gaps in coverage of HIV testing among people living with HIV in 16 African countries. Graham SM, editor. PLoS One. 2017;12(10):e0186316. 10.1371/journal.pone.0186316 PMC5638499 29023510

[12] Habib AG, Jumare J (2008). Migration, pastoralists, HIV infection and access to care: the nomadic Fulani of northern Nigeria. African J AIDS Res.

[13] Rhodes T (1994). HIV outreach, peer education and community change: developments and dilemmas. Health Educ J.

[14] Otiso L, McCollum R, Mireku M, Karuga R, de Koning K, Taegtmeyer M (2017). Decentralising and integrating HIV services in community-based health systems: a qualitative study of perceptions at macro, meso and micro levels of the health system. BMJ Glob Heal..

[15] Dieleman J, Campbell M, Chapin A, Eldrenkamp E, Fan VY, Haakenstad A, Kates J, Liu Y, Matyasz T, Micah A, Reynolds A, Sadat N, Schneider MT, Sorensen R, Evans T, Evans D, Kurowski C, Tandon A, Abbas KM, Abera SF, Kiadaliri AA, Ahmed KY, Ahmed MB, Alam K, Alizadeh-Navaei R, Alkerwi A', Amini E, Ammar W, Amrock SM, Antonio CAT, Atey TM, Avila-Burgos L, Awasthi A, Barac A, Bernal OA, Beyene AS, Beyene TJ, Birungi C, Bizuayehu HM, Breitborde NJK, Cahuana-Hurtado L, Castro RE, Catalia-Lopez F, Dalal K, Dandona L, Dandona R, de Jager P, Dharmaratne SD, Dubey M, Farinha CSS, Faro A, Feigl AB, Fischer F, Fitchett JRA, Foigt N, Giref AZ, Gupta R, Hamidi S, Harb HL, Hay SI, Hendrie D, Horino M, Jürisson M, Jakovljevic MB, Javanbakht M, John D, Jonas JB, Karimi SM, Khang YH, Khubchandani J, Kim YJ, Kinge JM, Krohn KJ, Kumar GA, el Razek HMA, el Razek MMA, Majeed A, Malekzadeh R, Masiye F, Meier T, Meretoja A, Miller TR, Mirrakhimov EM, Mohammed S, Nangia V, Olgiati S, Osman AS, Owolabi MO, Patel T, Caicedo AJP, Pereira DM, Perelman J, Polinder S, Rafay A, Rahimi-Movaghar V, Rai RK, Ram U, Ranabhat CL, Roba HS, Salama J, Savic M, Sepanlou SG, Shrime MG, Talongwa RT, Ao BJT, Tediosi F, Tesema AG, Thomson AJ, Tobe-Gai R, Topor-Madry R, Undurraga EA, Vasankari T, Violante FS, Werdecker A, Wijeratne T, Xu G, Yonemoto N, Younis MZ, Yu C, Zaidi Z, el Sayed Zaki M, Murray CJL (2017). Evolution and patterns of global health financing 1995-2014: development assistance for health, and government, prepaid private, and out-of-pocket health spending in 184 countries. Lancet..

[16] Chang AY, Cowling K, Micah AE, Chapin A, Chen CS, Ikilezi G, Sadat N, Tsakalos G, Wu J, Younker T, Zhao Y, Zlavog BS, Abbafati C, Ahmed AE, Alam K, Alipour V, Aljunid SM, Almalki MJ, Alvis-Guzman N, Ammar W, Andrei CL, Anjomshoa M, Antonio CAT, Arabloo J, Aremu O, Ausloos M, Avila-Burgos L, Awasthi A, Ayanore MA, Azari S, Azzopardi-Muscat N, Bagherzadeh M, Bärnighausen TW, Baune BT, Bayati M, Belay YB, Belay YA, Belete H, Berbada DA, Berman AE, Beuran M, Bijani A, Busse R, Cahuana-Hurtado L, Cámera LA, Catalá-López F, Chauhan BG, Constantin MM, Crowe CS, Cucu A, Dalal K, de Neve JW, Deiparine S, Demeke FM, Do HP, Dubey M, el Tantawi M, Eskandarieh S, Esmaeili R, Fakhar M, Fazaeli AA, Fischer F, Foigt NA, Fukumoto T, Fullman N, Galan A, Gamkrelidze A, Gezae KE, Ghajar A, Ghashghaee A, Goginashvili K, Haakenstad A, Haghparast Bidgoli H, Hamidi S, Harb HL, Hasanpoor E, Hassen HY, Hay SI, Hendrie D, Henok A, Heredia-Pi I, Herteliu C, Hoang CL, Hole MK, Homaie Rad E, Hossain N, Hosseinzadeh M, Hostiuc S, Ilesanmi OS, Irvani SSN, Jakovljevic M, Jalali A, James SL, Jonas JB, Jürisson M, Kadel R, Karami Matin B, Kasaeian A, Kasaye HK, Kassaw MW, Kazemi Karyani A, Khabiri R, Khan J, Khan MN, Khang YH, Kisa A, Kissimova-Skarbek K, Kohler S, Koyanagi A, Krohn KJ, Leung R, Lim LL, Lorkowski S, Majeed A, Malekzadeh R, Mansourian M, Mantovani LG, Massenburg BB, McKee M, Mehta V, Meretoja A, Meretoja TJ, Milevska Kostova N, Miller TR, Mirrakhimov EM, Mohajer B, Mohammad Darwesh A, Mohammed S, Mohebi F, Mokdad AH, Morrison SD, Mousavi SM, Muthupandian S, Nagarajan AJ, Nangia V, Negoi I, Nguyen CT, Nguyen HLT, Nguyen SH, Nosratnejad S, Oladimeji O, Olgiati S, Olusanya JO, Onwujekwe OE, Otstavnov SS, Pana A, Pereira DM, Piroozi B, Prada SI, Qorbani M, Rabiee M, Rabiee N, Rafiei A, Rahim F, Rahimi-Movaghar V, Ram U, Ranabhat CL, Ranta A, Rawaf DL, Rawaf S, Rezaei S, Roro EM, Rostami A, Rubino S, Salahshoor M, Samy AM, Sanabria J, Santos JV, Santric Milicevic MM, Sao Jose BP, Savic M, Schwendicke F, Sepanlou SG, Sepehrimanesh M, Sheikh A, Shrime MG, Sisay S, Soltani S, Soofi M, Soofi M, Srinivasan V, Tabarés-Seisdedos R, Torre A, Tovani-Palone MR, Tran BX, Tran KB, Undurraga EA, Valdez PR, van Boven JFM, Vargas V, Veisani Y, Violante FS, Vladimirov SK, Vlassov V, Vollmer S, Vu GT, Wolfe CDA, Yonemoto N, Younis MZ, Yousefifard M, Zaman SB, Zangeneh A, Zegeye EA, Ziapour A, Chew A, Murray CJL, Dieleman JL (2019). Past, present, and future of global health financing: a review of development assistance, government, out-of-pocket, and other private spending on health for 195 countries, 1995-2050. Lancet..

[17] OGAC. PEPFAR 3.0. Controlling the Epidemic: Delivering on the Promise of an AIDS-Free Generation. 2014.

[18] PEPFAR. FY 2015 Kenya Country Operational Plan 2016.

[19] PEPFAR. PEPFAR country/regional operational plan (COP/ROP) 2015 guidance. Washington, DC: PEPFAR; 2015.

[20] Marsh K, Eaton JW, Mahy M, Sabin K, Autenrieth CS, Wanyeki I, Daher J, Ghys PD (2019). Global, regional and country-level 90-90-90 estimates for 2018: assessing progress towards the 2020 target. Aids..

[21] National AIDS Control Council. Kenya HIV County Profiles. Nairobi, Kenya: NACC, Ministry of Health, Government of Kenya. 2014. Available from: http://nacc.or.ke/wp-content/uploads/2016/12/Kenya-HIV-County-Profiles-2016.pdf%0Ahttp://www.nacc.or.ke/attachments/article/464/KenyaCountyProfilesBook_Nov_print.pdf.

[22] Health Policy Project. Health Financing Profile: Kenya. Washington, DC: Futures Group; 2016. Available from: https://www.healthpolicyproject.com/pubs/7887/Kenya_HFP.pdf

[23] NACC. Kenya AIDS Strategic Framework (KASF) 2015. 2015. Available from: http://nacc.or.ke/wp-content/uploads/2015/09/KASF_Final.pdf

[24] Haider H. Conflict Analysis of North Eastern Kenya. Brighton, UK; 2020. (K4D Emerging Issues). Report No.: 36. Available from: https://opendocs.ids.ac.uk/opendocs/handle/20.500.12413/15570

[25] Juliastuti D, Dean J, Fitzgerald L (2020). Sexual and reproductive health of women living with HIV in Muslim-majority countries: a systematic mixed studies review. BMC Int Health Hum Rights.

[26] PWC. Africa Gearing Up. 2013.

[27] Deloitte. Kenya: Grounding Africa’s Economic Growth. 2016.

[28] Deane KD, Parkhurst JO, Johnston D. Linking migration, mobility and HIV. Trop Med Int Heal 2010;15(12):1458–1463. Available from: http://doi.wiley.com/10.1111/j.1365-3156.2010.02647.x 10.1111/j.1365-3156.2010.02647.x 20958895

[29] McCollum R, Theobald S, Otiso L, Martineau T, Karuga R, Barasa E, Molyneux S, Taegtmeyer M (2018). Priority setting for health in the context of devolution in Kenya: implications for health equity and community-based primary care. Health Policy Plan.

[30] Wilhelm JA, Qiu M, Paina L, Colantuoni E, Mukuru M, Ssengooba F, et al. The impact of PEPFAR transition on HIV service delivery at health facilities in Uganda. Rockers P, editor. PLoS One. 2019;14(10):e0223426. 10.1371/journal.pone.0223426 PMC6785126 31596884

[31] PEPFAR. Uganda Country / Regional Operational Plan 2016. 2017;1–65.

[32] UNAIDS. Uganda: Developing Subnational Estimates of HIV Prevalence and the Number of People Living With HIV. Geneva: UNAIDS; 2014.

[33] Koseki S, Fagan T, Menon V. Sustainable HIV Financing in Uganda. Washington, D.C.; 2015.

[34] Rodríguez DC, Mohan D, Mackenzie C, Wilhelm J, Eze-Ajoku E, Omondi E, et al. Effects of transition on HIV and non-HIV services and health systems in Kenya: a mixed methods evaluation of donor transition. BMC Health Serv Res [Internet]. 2021;21(1):457. Available from: https://bmchealthservres.biomedcentral.com/articles/10.1186/s12913-021-06451-y. 10.1186/s12913-021-06451-y PMC8117613 33985482

[35] Bennett S, Singh S, Rodriguez D, Ozawa S, Singh K, Chhabra V, et al. Transitioning a Large Scale HIV/AIDS Prevention Program to Local Stakeholders: Findings from the Avahan Transition Evaluation. Lima VD, editor. PLoS One. 2015;10(9):e0136177. 10.1371/journal.pone.0136177 PMC4556643 26327591

[36] Banigbe B, Audet CM, Okonkwo P, Arije OO, Bassi E, Clouse K, et al. Effect of PEPFAR funding policy change on HIV service delivery in a large HIV care and treatment network in Nigeria. PLoS One [Internet]. 2019;14(9):1–12. Available from: 10.1371/journal.pone.0221809. 10.1371/journal.pone.0221809 PMC6760763 31553735

[37] Gerring J (2004). What is a case study and what is it good for?. Am Polit Sci Rev..

[38] Bennett S, Paina L, Rodriguez D, Wilhelm JA, Mackenzie C, Mukuru M, et al. Project SOAR Report: Evaluating the Impact of PEPFAR’s Geographic Prioritization on Centrally-Supported Health Facilities. Baltimore; 2018.

[39] Wilhelm JA, Paina L, Qiu M, Zakumumpa H, Bennett S. The differential impacts of PEPFAR transition on private for-profit, private not-for-profit and publicly owned health facilities in Uganda. Health Policy Plan. 2020;35(2):133–41. 10.1093/heapol/czz090. 10.1093/heapol/czz090 PMC7050684 31713608

[40] World Health Organization. Operations Manual for Delivery of HIV Prevention, Care and Treatment at Primary Health Centres in High-Prevalence, Resource-Constrained Settings. Geneva: WHO; 2008. 26290930

[41] Okeke NL, Ostermann J, Thielman NM (2014). Enhancing linkage and retention in HIV care: a review of interventions for highly resourced and resource-poor settings. Curr HIV/AIDS Rep..

[42] Sharma M, Ying R, Tarr G, Barnabas R (2015). Systematic review and meta-analysis of community and facility-based HIV testing to address linkage to care gaps in sub-Saharan Africa. Nature..

[43] Coates TJ, Kulich M, Celentano DD, Zelaya CE, Chariyalertsak S, Chingono A (2014). Effect of community-based voluntary counselling and testing on HIV incidence and social and behavioural outcomes (NIMH project accept; HPTN 043): a cluster-randomised trial. Lancet Glob Heal.

[44] Bemelmans M, Baert S, Goemaere E, Wilkinson L, Vandendyck M, van Cutsem G, Silva C, Perry S, Szumilin E, Gerstenhaber R, Kalenga L, Biot M, Ford N (2014). Community-supported models of care for people on HIV treatment in sub-Saharan Africa. Trop Med Int Heal.

[45] Sabapathy K, van den Bergh R, Fidler S, Hayes R, Ford N (2012). Uptake of home-based voluntary HIV testing in Sub-Saharan Africa: a systematic review and meta-analysis. PLoS Med..

[46] Mwango LK, Stafford KA, Blanco NC, Lavoie MC, Mujansi M, Nyirongo N (2020). Index and targeted community-based testing to optimize HIV case finding and ART linkage among men in Zambia. J Int AIDS Soc.

[47] Petersen M, Balzer L, Kwarsiima D, Sang N, Chamie G, Ayieko J, Kabami J, Owaraganise A, Liegler T, Mwangwa F, Kadede K, Jain V, Plenty A, Brown L, Lavoy G, Schwab J, Black D, van der Laan M, Bukusi EA, Cohen CR, Clark TD, Charlebois E, Kamya M, Havlir D (2017). Association of Implementation of a universal testing and treatment intervention with HIV diagnosis, receipt of antiretroviral therapy, and viral suppression in East Africa. JAMA..

[48] Sabin LL, Semrau K, Desilva M, Le LTT, Beard JJ, Hamer DH (2019). Effectiveness of community outreach HIV prevention programs in Vietnam: a mixed methods evaluation. BMC Public Health.

[49] Geng EH, Nash D, Kambugu A, Zhang Y, Braitstein P, Christopoulos KA, Muyindike W, Bwana MB, Yiannoutsos CT, Petersen ML, Martin JN (2010). Retention in care among HIV-infected patients in resource-limited settings: emerging insights and new directions. Curr HIV/AIDS Rep.

[50] Gusdal AK, Obua C, Andualem T, Wahlström R, Chalker J, Fochsen G, on behalf of the INRUD-IAA project (2011). Peer counselors’ role in supporting patients’ adherence to ART in Ethiopia and Uganda. AIDS Care.

[51] Zachariah R, Teck R, Buhendwa L, Fitzerland M, Labana S, Chinji C, Humblet P, Harries AD (2007). Community support is associated with better antiretroviral treatment outcomes in a resource-limited rural district in Malawi. Trans R Soc Trop Med Hyg.

[52] Kabore I, Bloem J, Etheredge G, Obiero W, Wanless S, Doykos P, Ntsekhe P, Mtshali N, Afrikaner E, Sayed R, Bostwelelo J, Hani A, Moshabesha T, Kalaka A, Mameja J, Zwane N, Shongwe N, Mtshali P, Mohr B, Smuts A, Tiam A (2010). The effect of community-based support services on clinical efficacy and health-related quality of life in HIV/AIDS patients in resource-limited settings in sub-Saharan Africa. AIDS Patient Care STDs.

[53] Thomson KA, Cheti EO, Reid T (2011). Implementation and outcomes of an active defaulter tracing system for HIV, prevention of mother to child transmission of HIV (PMTCT), and TB patients in Kibera, Nairobi, Kenya. Trans R Soc Trop Med Hyg.

[54] World Health Organization. Technical and operational considerations for implementing HIV viral load testing: Interim technical update. Geneva: WHO; 2014.

[55] Marcus R, Ferrand RA, Kranzer K, Bekker L-G (2017). The case for viral load testing in adolescents in resource-limited settings. J Int AIDS Soc.

[56] Rodríguez DC, Paina L, Wilhelm J, Mackenzie C, Mukuru M, Ssengooba F, et al. Evaluating the impact of PEPFAR’s geographic prioritization on centrally supported health facilities. Washington, D.C: Population Council; 2019. Available from: http://www.projsoar.org/wp-content/uploads/2019/03/KenyUg_PEPFARGeo_Report.pdf

[57] Vogus A, Graff K (2015). PEPFAR transitions to country ownership: review of past donor transitions and application of lessons learned to the eastern Caribbean. Glob Heal Sci Pract.

[58] Stierman E, Ssengooba F, Bennett S (2013). Aid alignment: a longer term lens on trends in development assistance for health in Uganda. Glob Health.

[59] Grépin KA. HIV donor funding has both boosted and curbed the delivery of different non-HIV health services in sub-Saharan Africa. Health Aff 2012;31(7):1406–1414. Available from: http://www.healthaffairs.org/doi/10.1377/hlthaff.2012.0279 10.1377/hlthaff.2012.0279 22778329

